# Association of muscle and visceral adipose tissues with the probability of Alzheimer’s disease in healthy subjects

**DOI:** 10.1038/s41598-018-37244-9

**Published:** 2019-01-30

**Authors:** Jahae Kim, Kang-Ho Choi, Sang-Geon Cho, Sae-Ryung Kang, Su Woong Yoo, Seong Young Kwon, Jung-Joon Min, Hee-Seung Bom, Ho-Chun Song

**Affiliations:** 1 Department of Nuclear Medicine, Chonnam National University Hospital, Jebongro 42, Donggu, Gwangju, Republic of Korea; 2 Department of Neurology, Chonnam National University Hospital, Jebongro 42, Donggu, Gwangju, Republic of Korea

## Abstract

Increasing evidence indicates that sarcopenia and obesity can be risk factors for incident dementia. We investigated the association of body composition including muscle and visceral adipose tissue (VAT) with the probability of Alzheimer’s disease (AD) in healthy middle-aged and elderly subjects using ^18^F-fluorodeoxyglucose (FDG) positron emission tomography (PET)/computed tomography (CT). This study included 110 healthy subjects with available whole-body FDG PET/CT scans and medical records. Muscle and VAT tissues were measured on the abdominal CT slice, and the PMOD Alzheimer’s discrimination FDG PET analysis tool (PALZ) score was evaluated on the brain PET of the same subject using software PALZ. Skeletal muscle index (r: −0.306; *P* = 0.031) was significantly negatively associated with the PALZ score in the elderly patients. Muscle area (β: −0.640; *P* = 0.043) and skeletal muscle index (β: −0.557; *P* = 0.043) were independently associated with the PALZ score in elderly subjects after adjustments for sex, duration of education, hypertension, diabetes mellitus, and smoking and drinking status. Increased muscle tissue was associated with a lower probability of AD in elderly subjects, but VAT was not associated with a lower probability of AD in middle-or older-aged adults.

## Introduction

A number of lifestyle factors such as physical activity, diet, intellectual engagement, and social interaction are associated with the risk of age-associated neurodegenerative disorders, such as Alzheimer’s disease (AD) and vascular dementia^1^. Recent publications have suggested that sarcopenia and obesity can also be risk factors for incident dementia. Sarcopenia is a loss of muscle mass and muscle strength, and body weight with advancing aging. Men lose muscle mass at a rate of 0.5% to 1.0% per year after the age of 70 years, and women a rate of 3.7% per decade^2^. Previous studies have demonstrated that low muscle mass is associated with cognitive impairment^3–5^. Several studies have been performed to determine whether obesity is a risk factor for incident dementia. Obesity has a positive correlation with dementia in middle-aged adults, but a negative correlation with dementia in aged adults^6^. Studies have suggested that mid-life obesity is associated with a greater risk of subsequent dementia^7–9^, but obesity in late-life is associated with a decreased likelihood of dementia^10–12^.

^18^F-fluorodeoxyglucose (FDG) positron emission tomography (PET) has been used as a neurodegenerative biomarker for AD^13–15^. Glucose metabolic reductions on FDG PET precede the onset of clinical symptoms and a pattern of glucose metabolic reduction in the parietotemporal and posterior cingulate cortices offers *in vivo* temporal and topographic information on the development of AD^15^. In contrast, computed tomography (CT), which is often used in conjunction with FDG-PET in the integrated PET/CT system is considered a gold standard for the assessment of body composition^16^. Bone, skeletal muscle and adipose tissue have specific Hounsfield unit ranges on a CT scan, which enables their identification. Moreover, several studies have confirmed that whole-body fat and lean tissue mass can be estimated using single images at key lumbar vertebral landmarks^17^.

In this retrospective study, we used FDG PET/CT scans to determine the association between body composition and the risk of AD in healthy subjects. We measured body composition, including muscle and visceral adipose tissue on the CT scan. We then assessed the risk of AD by assessing cerebral glucose hypometabolism on brain FDG PET scans. To the best of our knowledge, this is the first study to present evidence of the potential value of FDG PET/CT scans for this purpose.

## Results

The clinical characteristics of the subjects are listed in Table 1. A total of 110 subjects had a mean ( ± SD) age of 63.0 ± 6.4 years. Sixty-five subjects (59%) were men and 45 (41%) were women. There were 60 (55%) subjects in the mid-life group and 50 (45%) in the late-life group. The late-life group had a higher proportion (n = 26, 52%) of hypertension than the mid-life group; the mid-life group had a higher proportion (n = 28, 47%) of drinking habits than the late-life group. There was no significant difference in anthropometric variables between the groups, but the mean muscle HU was higher in the mid-life group than in the late-life group (*P* = 0.018) among the variables for body composition. The MMSE score was higher in the mid-life group than in the late-life group (*P* = 0.007).

**Table 1:** Characteristics of study subjects.

	All	Midlife (<60 years)	Late-life (≥60 years)	*P*
**Baseline characteristics**				
Age (y)	63.0 ± 6.4	55.6 ± 2.8	65.4 ± 4.7	<0.001
Sex, male	65 (59%)	31 (52%)	34 (68%)	0.119
Hypertension	41 (37%)	15 (25%)	26 (52%)	0.005
Diabetes mellitus	11 (10%)	4 (7%)	7 (14%)	0.221
Smoking	18 (16%)	11 (18%)	7 (14%)	0.611
Drinking	39 (36%)	28 (47%)	11 (22%)	0.009
**Anthropometric measures of body composition**				
Body weight (kg)	66.2 ± 11.6	65.3 ± 12.4	67.4 ± 10.4	0.354
Height (cm)	163.5 ± 8.6	162.8 ± 9.1	164.4 ± 7.8	0.322
BMI	24.7 ± 3.1	24.5 ± 3.2	24.9 ± 2.9	0.529
**CT measures of body composition**				
Muscle area (cm^2^)	156.9 ± 37.8	153.7 ± 42.4	160.7 ± 31.5	0.333
Muscle HU	35.5 ± 4.7	36.5 ± 4.9	34.3 ± 4.3	0.018
Skeletal muscle index	58.1 ± 10.7	57.3 ± 12.2	59.1 ± 8.5	0.385
VAT area (cm^2^)	132.1 ± 62.9	132.3 ± 67.4	131.8 ± 57.6	0.965
VAT HU	−94.7 ± 5.8	−95.4 ± 5.9	−93.8 ± 5.6	0.148
VAT index	49.0 ± 21.8	49.1 ± 23.0	48.9 ± 20.6	0.957
Muscle to VAT ratio	1.5 ± 1.0	1.4 ± 0.7	1.6 ± 1.2	0.449
**AD-related characteristics**				
Education (y)	9.9 ± 3.3	10.5 ± 2.7	9.2 ± 3.7	0.106
MMSE	27.7 ± 2.2	28.3 ± 1.8	27.1 ± 2.5	0.007
PALZ score	3630.2 ± 1737.9	3518.1 ± 1677.6	3764.8 ± 1815.6	0.461

M, male; F, female; y, years; BMI, body mass index; HU, Hounsfield unit; VAT, visceral adipose tissue; AD, Alzheimer’s disease; MMSE, mini-mental state examination; PALZ, PMOD Alzheimer’s discrimination.

**Figure 1:**
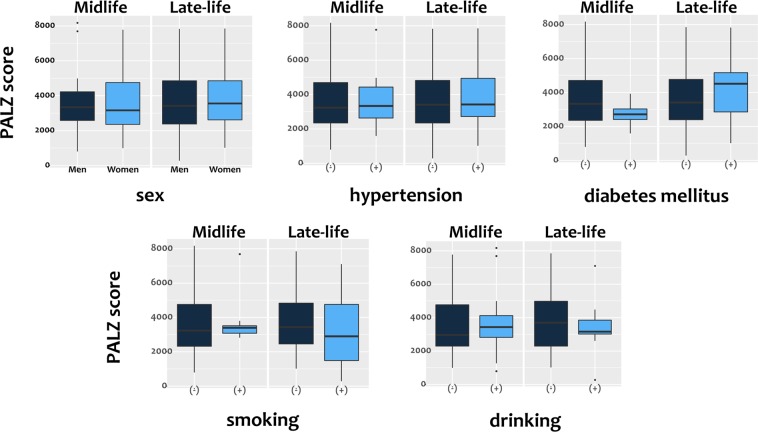
Box plots of the PALZ scores of the midlife and late-life groups according to clinical characteristics. The PALZ score differences according to sex, history of hypertension or diabetes mellitus, and smoking and drinking habits in between the mid-life and late-life groups were not significant (all *P* > 0.05). PALZ; PMOD Alzheimer’s discrimination.

A correlation analysis between the CT and anthropometric variables of body composition with the PMOD Alzheimer’s discrimination analysis tool (PALZ) score (Table 2) revealed no significant association. In the subgroup analysis according to age, there was also no significant correlation between the CT and anthropometric variables with the PALZ score in the mid-life group. Skeletal muscle index (r: −0.306; *P* = 0.031) was significantly negatively associated with the PALZ score in the late-life group. Table 2 shows the correlations between the PALZ score and adipose tissue and skeletal muscle indices of both groups. There was no significant correlation between the PALZ score and adipose tissue or skeletal muscle indices in the mid-life group. In the late-life group, there was no significant correlation between the PALZ score and the adipose tissue index. However, the skeletal muscle index showed a significant correlation with the PALZ score; higher skeletal muscle index was associated with a lower PALZ score. There were no differences in the PALZ score, in either the mid or late-life group, with respect to sex, history of hypertension or diabetes mellitus, smoking, or drinking (Fig. 1). Moreover, there was no significant correlation between MMSE and PALZ score in both the mid-life group (r: 0.096, *P* = 0.516) and late-life group (r: 0.061, *P* = 0.687).

**Table 2:** Analysis of the correlation between body composition and anthropometric variables associated with the PALZ score.

	All		Midlife (<60 years)		Late-life (≥60 years)	
	Pearson’s r	*P*	Pearson’s r	*P*	Pearson’s r	*P*
**Body composition measures**						
Muscle area (cm^2^)	−0.112	0.244	−0.022	0.867	−0.271	0.057
Muscle HU	−0.046	0.632	−0.018	0.889	−0.048	0.743
Skeletal muscle index	−0.151	0.115	−0.071	0.590	−0.306	0.031
VAT area (cm^2^)	0.027	0.782	0.097	0.460	−0.064	0.660
VAT HU	−0.054	0.575	−0.099	0.450	−0.024	0.868
VAT index	0.014	0.887	0.064	0.627	−0.048	0.742
Muscle to VAT ratio	−0.063	0.515	0.038	0.772	−0.146	0.312
**Anthropometric measures**						
Body weight (kg)	−0.084	0.383	0.011	0.933	−0.228	0.112
Height (cm)	0.030	0.759	0.094	0.473	−0.069	0.635
BMI (kg/m^2^)	−0.141	0.141	−0.075	0.568	−0.235	0.100

HU, Hounsfield unit; VAT, visceral adipose tissue; BMI, body mass index.

**Figure 2:**
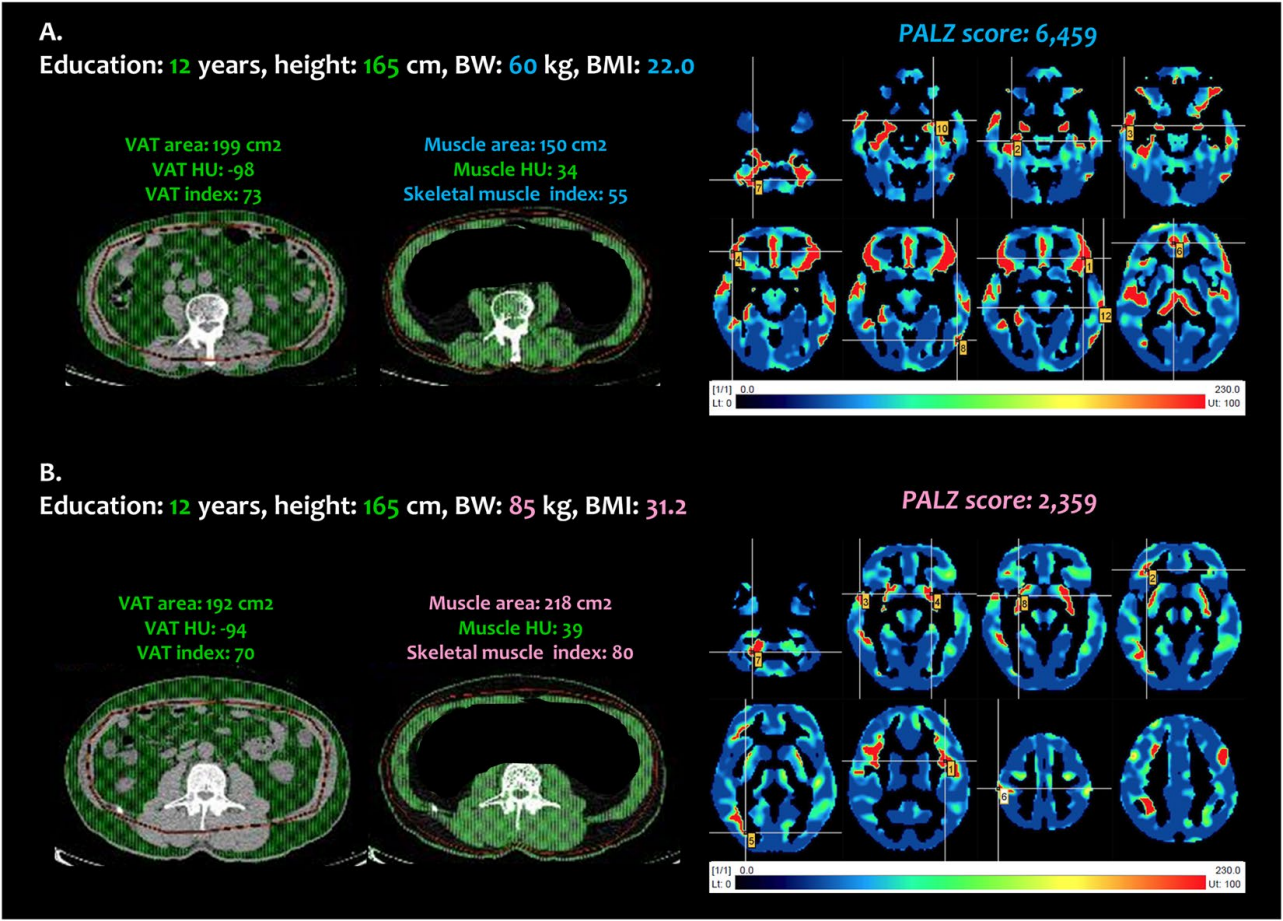
Representative cases. (**A**) A healthy 72-year-old man (education: 12 years, height: 165 cm, body weight: 60 kg, BMI: 22.0 kg/m^2^), (**B**) A healthy 73-year-old man (education: 12 years, height: 165 cm, body weight: 85 kg, BMI: 31.2 kg/m^2^). BMI, body mass index; VAT, visceral adipose tissue; HU, Hounsfield unit; PALZ, PMOD Alzheimer’s discrimination.

To determine the factors associated with the PALZ score in the late-life group, a multiple linear regression analysis was performed while adjusting for sex, duration of education, and/or potential confounding factors (hypertension, diabetes mellitus, and smoking and drinking habits). Due to multicollinearity between the CT variables of body composition, each variable was entered into the multivariate model separately. Linear regression analysis showed that the muscle area and skeletal muscle index were significantly negatively associated with PALZ score after adjusting for confounding factors in both Model 1 and Model 2 (Table 3). In the late-life group, subjects with a lower muscle area and skeletal muscle index had a higher PALZ score (Fig. 2A), whereas subjects with a higher muscle area and skeletal muscle index had a lower PALZ score (Fig. 2B).

**Table 3:** Linear regression analysis associated with PALZ score in the late-life group.

	Model 1			Model 2		
	β	*P*	95% CI	β	*P*	95% CI
Muscle area (cm^2^)	−0.629	0.021	−0.708–−0.063	−0.640	0.043	−0.770–−0.014
Skeletal muscle index	−0.472	0.032	−204.450–−9.904	−0.557	0.043	−248.247–−4.556
VAT area (cm^2^)	−0.010	0.958	−0.128–0.121	0.003	0.987	−0.137–0.139
VAT index	−0.006	0.973	−33.911–32.784	0.003	0.988	−37.044–37.584

Model 1 was adjusted for sex and education; Model 2 was adjusted for the variables in Model 1 plus HTN, DM, and smoking and drinking.

β, standardized coefficient value; CI, confidence interval; HTN, hypertension; DM, diabetes mellitus.

## Discussion

In this study, we found that the skeletal muscle index was associated with the probability of AD on ^18^F-FDG PET/CT in the late-life group. A higher amount of muscle tissue was associated with a lower probability of AD in the elderly. These results are similar to those of previous epidemiologic studies that found that physical activities are beneficial for cognitive function in healthy older adults^18,19^. Erickson *et al*. showed that higher levels of aerobic fitness are associated with increased hippocampal volume in older adults^20^. Colcombe *et al*. showed that physical fitness is associated with reduced loss of hippocampal brain tissue in the aging brain^21^. These studies demonstrated the association between physical function and hippocampal volume on brain MRI, which is one AD biomarker^14^. Other studies have also demonstrated that physical activity is associated with a reduction of the risk of age-associated neurodegenerative disorders such as AD^22–24^. Physical activity is defined as any bodily movement produced by skeletal muscles that expend energy; a subset of physical activity, such as resistance exercise, increases muscle size in the elderly^25^. The assessment of physical activity varied with the type of activity, the exercise training program, and duration in the previous studies. However, skeletal muscle mass, which is an objective, clear, and reproducible index using CT scanning, was used in this study to assess physical activity. In addition, decreased FDG uptake on PET is an earlier biomarker of neuronal injury, dysfunction, and degeneration than structural MRI, the last biomarker^26^. The t-sum, which is computed as the PALZ score by PMOD software, is a global index of AD-related hypometabolism^27^. Therefore, the findings of this study are in accordance with results of previous studies, and this study supports and specifies the relationship between skeletal muscle and cognitive function in the elderly by assessing the skeletal muscle mass on CT scans and the cerebral glucose metabolism on PET scans.

In contrast to previous reports, we found that no significant correlation between the VAT and the probability of AD. Previous reports have suggested that obesity increases the risk of dementia development^28–31^. Several factors might explain the difference between our study and previous studies. First, we used more accurate, adiposity measures to assess obesity. Previous studies used a simple measure of adiposity such as BMI, waist circumference, or waist-to-hip ratio to assess adiposity. BMI is a widely used measure of obesity, but it does not differentiate between lean mass and fat mass and does not consider fat distribution^32^. Waist circumference and the waist-to-hip ratio are also not good indicators of abdominal adiposity due to over- and under-evaluation of the risk in patients of different heights. However, CT measurement can directly visualize visceral adiposity that is more closely related to metabolic complications of obesity irrespective of age, gender, ethnicity, and height^33^. Although CT is not recommended for the routine evaluation of VAT due to radiation exposure, this retrospective study was performed using previous PET/CT scans. Therefore, it was possible to evaluate the relationship between visceral adipose tissue and the probability of AD without exposing the subjects to additional radiation. Our results showed that the relations between visceral adiposity measured on CT scans and the probability of AD were not significant in healthy elderly subjects. As each measure of adiposity showed a different fat mass, further studies are necessary to elucidate which measure of adiposity helps define the risk of AD development.

Second, we used an earlier biomarker for the assessment of the risk of AD. Previous studies used biomarkers that are detectable later in AD progression, such as the neuropsychiatric test score and global or hippocampal atrophy on MRI^34,35^. AD biomarkers become abnormal in a temporally ordered manner. FDG PET is earlier than structural MRI, and these biomarker abnormalities precede clinical symptoms^14^. Therefore, we believe that our method of cerebral glucose metabolism on FDG PET has advantages over those previously used to evaluate the risk of AD in the earlier stage of AD progress. Most previous studies reporting that obesity increases the risk of AD^7,36,37^, and its association in healthy individuals has been demonstrated in the Volkow *et al*.’s study using FDG PET^38^. They revealed an association between higher BMI and lower prefrontal metabolic activity, which contribute to the impairment of cognitive function. However, some studies have reported the lack of correlation between obesity and AD risk. Gustafson *et al*. reported no relationship between excess adiposity and the gray matter volume of the frontal, parietal, or occipital lobes; there was a slight relationship with a small significance in the temporal lobes^39^. Soriano-Maldonado *et al*. also suggested that there is no association between central adiposity and cognitive dysfunction in patients with fibromyalgia^40^. Of the two body composition parameters addressed in this study, there is a consistent association between skeletal muscle and the risk of AD, whereas conflicting results have been observed in studies on the association between obesity and the risk of AD in elderly subjects^41,42^. The relationship between VAT and the risk of AD has not yet been fully determined; therefore, further studies are necessary.

This study had several limitations. First, it had a small sample size and a cross-sectional design. Previous studies of the risk of AD using this imaging modality enrolled a larger number of subjects. A longitudinal study is required to elucidate the actual relationship between body composition and the risk of AD. Second, this study had a retrospective design. Prospective studies will be necessary, but this retrospective study will be useful because a retrospective review does not have to consider the additional radiation exposure due to FDG PET/CT. Partial CT scans for screening body composition with effective dose under 1 mSv or other measures without risk of radiation exposure such as MRI or ultrasound may be preferred in future prospective studies. Third, care must be taken not to overinterpret the risk of AD using the PALZ index in the healthy cohort, because t-sum higher than 11,090 (or 13,341^43^) are usually regarded as abnormal. Fourth, this study might have a selection bias. All enrolled subjects visited the Health Promotion Center at our institution. They were concerned about their health and interested in a health care program. This group, with a high social status, might not be generalizable to the entire healthy population. Finally, other AD risk factors such as APOE4 could not be evaluated in this study.

## Conclusion

The present study showed increased muscle tissue was associated with a lower probability of AD in the elderly, but visceral adiposity was not associated with a lower probability of AD in middle-aged or elderly subjects. This study shows that it is important to increase the skeletal muscle mass to decrease the risk of AD in the elderly. This is the first study that evaluated the association between body composition and the risk of AD using ^18^F-FDG PET/CT.

## Methods

### Study population

Subjects who visited the Health Promotion Center in our hospital from 2009 to 2014 for medical check-ups related to disease prevention were selected for inclusion in this study. From these subjects, 110 cases were eligible for retrospective analysis. The primary inclusion criteria were as follows: (1) brain FDG PET/CT scan available, (2) brain MRI performed within 1 year of the PET/CT scan, and (3) mini-mental state examination (MMSE) conducted by well-trained medical doctors on the same day as the PET/CT. Exclusion criteria were as follows: (1) evidence of brain tumors, major infarctions and hemorrhages on the brain MRI scan, (2) cognitive impairment, as indicated by MMSE scores below the 16^th^ percentile for age- and education-matched norms, (3) history or clinically suspected anxiety or depression symptoms, and (4) failure of PMOD software analysis or an abnormal PMOD Alzheimer’s discrimination (PALZ) score (≥11,090)^44^. This retrospective analysis was ethically approved by our institutional review board of the Chonnam National University Hospital (#2017-107), and the requirement to obtain informed consent was waived. All procedures were performed in accordance with relevant guidelines and regulations.

Baseline demographic and clinical information were collected from all study subjects and included the following: age, sex, history of hypertension (previous use of antihypertensive medication, systolic blood pressure >140 mm Hg, or diastolic blood pressure >90 mm Hg), diabetes mellitus (previous use of glucose-lowering medication or fasting blood sugar level ≥126 mg/dL), smoking (current or ex-smokers) and drinking (1 drink or more per week) habits, and body weight and height. The body mass index was calculated as body mass in kilograms divided by height in meters squared. The duration of education was learned from the interview for MMSE. For the group comparison according to age, a cut-off of 60 years was proposed for differentiation between mid-life (<60 years) and late-life (≥60 years). All data generated or analysed during this study are included in this published article.

### Image acquisition

All subjects fasted for at least 6 hours before the procedure and their blood glucose was <160 mg/dL at the time of the scan. Subjects were injected with 4.8 MBq/kg of ^18^F-FDG, and then rested in a quiet, dimly lit room for 50 min to 1 h. PET/CT studies were performed using a combined PET/CT scanner (Discovery STE System; GE Medical Systems, Milwaukee, WI, USA). The CT scan was first obtained for attenuation correction with a peak voltage of 120 kVp, automated tube current from 10 to 130 mA, and slice thickness of 3.75 mm from the top of the skull to the thigh. An emission scan was obtained of the torso region from the skull base to the proximal thigh with 7 to 8 beds (16 cm per bed with overlap of 4 cm), where the time per bed was 150 seconds. The brain scan was performed in a single bed (15 cm per bed) for 5 min. Brain PET images were reconstructed using an ordered subset expectation maximization (OSEM) algorithm. Attenuation correction was based on the CT scan and scatter correction was performed using standard software as supplied by the scanner manufacturer.

### Image analysis

PET and CT images were assessed by an experienced nuclear medicine physician who was blinded to the clinical information.

### PET analysis and PALZ score calculation

Multiplanar images were displayed and analyzed with PMOD software v. 2.9 (PMOD Technologies Ltd, Zurich, Switzerland). To evaluate AD-related hypometabolism, the PALZ score^45^, which is a global measure of PET scan abnormalities, was computed using the “Alzheimer” option in the PMOD software. Individual FDG PET images were compared to a reference database of normal scans of patients >50 years of age using a voxel-wise *t*-test, based on the subject’s age and the voxel-dependent age-regression parameters. The PALZ score is defined as the sum of all *t*-values of voxels with an FDG uptake below the 95% age-adjusted prediction limit within a pre-defined AD-pattern mask^46^. Given the parameters from statistical maps identifying voxels with abnormal metabolism can be extracted that reflect the overall severity of metabolic deficits which represents the total metabolic impairment in areas typically affected by AD, so a higher PALZ score indicated an increased probability of AD, and 11,090 was set for the threshold of abnormality^47^. All PET images were checked to ensure proper implementation of normalization and fusion methods provided by the PALZ.

### CT analysis

CT images were analyzed on an Advantage 4.6 workstation (GE Medical Systems). CT analysis was conducted at the third lumbar vertebra (L3), which is the landmark of interest for the CT studies of body composition, because the single abdominal cross-sectional image at L3 was the best correlate of whole-body composition^17^. The CT display contrast window was from −29 to 150 Hounsfield units (HU) for skeletal muscle and from −150 to −50 HU for adipose tissue. To quantify skeletal muscle, an ROI was drawn by tracing along the fascial plane, including muscles such as the rectus abdominis, abdominal, psoas, and paraspinal muscles. To separate the internal organs from pure muscle, the areas including internal organs such as the kidneys, vessels, and small bowel loops were subtracted from the ROI. Three skeletal muscle measurements were derived: (1) muscle area, which was defined as the cross-sectional area of muscle at the L3 level, (2) muscle HU, which was defined as mean muscle attenuation, and (3) the skeletal muscle index, which was defined as the muscle area normalized for height in meters squared. To quantify the visceral adipose tissue (VAT), another ROI defining the internal abdominal wall was drawn, as described previously^48^. Three VAT measures were derived: (1) the VAT area, which was defined as the cross-sectional area of visceral adipose tissue at the L3 level, (2) VAT HU, which was defined as mean visceral adipose tissue attenuation, and (3) the VAT index, which was defined as the VAT area normalized for height in meters squared.

### Statistical analysis

Continuous variables were expressed as the mean ± standard deviation (SD); categorical variables were expressed as frequencies and percentages. Differences between groups were compared using a Student’s t-test or Mann-Whitney test for continuous variables and a Fisher’s exact test for categorical variables. Correlations between continuous variables were assessed using the Pearson correlation coefficient. Two different linear regression analyses were used to analyze the association between body composition measures and the PALZ score. Model 1 included adjustments for sex and the duration of education. Model 2 included potential confounding variables such as hypertension, diabetes mellitus, and smoking and drinking habits. Age was not included, because the PALZ score resulted from the adjustment of age as a confounding variable. Statistical analysis was performed using IBM SPSS Statistics for Windows, Version 21.0 (IBM Corp., Armonk, NY, USA), and boxplots were drawn using an open-source package for R.
